# Risk assessment of morbidities after right hemicolectomy based on the National Clinical Database in Japan

**DOI:** 10.1002/ags3.12067

**Published:** 2018-04-16

**Authors:** Takahiro Yoshida, Hiroaki Miyata, Hiroyuki Konno, Hiraku Kumamaru, Akira Tangoku, Yoshihito Furukita, Norimichi Hirahara, Go Wakabayashi, Mitsukazu Gotoh, Masaki Mori

**Affiliations:** ^1^ The Japanese Society of Gastroenterological Surgery Database Committee Working Group Tokyo Japan; ^2^ The Japanese Society of Gastroenterological Surgery Tokyo Japan; ^3^ National Clinical Database Tokyo Japan

**Keywords:** Japanese National Clinical Database, morbidity, right hemicolectomy, risk model

## Abstract

**Objective:**

Nationwide databases are expected to provide critical data to improve medical practice. The present study used such data to develop risk models for clinically important outcomes after right hemicolectomy based on preoperative risk factors.

**Methods:**

Japan's National Clinical Database (NCD) identified 38 030 cases of right hemicolectomy in the years 2011 and 2012. These were used to analyze correlations between mortality and eight selected clinical outcomes of interest by Pearson's correlation coefficient (*r*). To construct risk models for the eight selected clinical outcomes, 80% of all the examined cases were extracted randomly as a development cohort, and preoperative risk factors for each clinical outcome were identified using a forward stepwise selection method. Morbidities predicted from the risk models were used to find areas under the receiver operator curves among the remaining 20% of the testing cohort.

**Results:**

The following clinical outcomes were identified as highly associated with operative mortality: systemic sepsis (*r* = .360), renal failure (*r* = .341), unplanned intubation (*r* = .316) and central nervous system (CNS) occurrences (*r* = .301). Risk models containing up to 21 preoperative variables were constructed for these eight postoperative clinical outcomes. Predictive values of the eight models were as follows: surgical site infections (0.634), anastomotic leakage (0.656), systemic sepsis (0.816), pneumonia (0.846), unplanned intubation (0.838), renal failure (0.883), CNS occurrences (0.833) and transfusion >5 units (0.846).

**Conclusions:**

This study indicated that the NCD‐generated risk models for six of the eight selected critical postoperative outcomes had high discrimination among right hemicolectomy patients. These risk models can accurately identify high‐risk patients prior to surgery.

## Abstract 2

This article is the first study that shows risk models for eight clinically important factors for morbidity after right hemicolectomy, based on preoperative risk factors. Our models and their evaluations are based on data from the NCD in Japan for the years 2011 and 2012.

## 1 INTRODUCTION

Nationwide medical databases are expected to improve clinical outcomes at relevant institutions. For instance, the US Veterans Health Administration (VHA) established the American College of Surgeons (ACS) National Surgical Quality Improvement Program (NSQIP) in 1994 for monitoring and improving the quality of surgical care across all VHA medical centers in the USA.^1^ This program demonstrably enhanced the quality of surgical care.^2^

In Japan, the National Clinical Database (NCD) was founded in April 2010 as the parent body of the database system linked to the board certification system. Registration began in 2011 and, to date, more than 4600 facilities have enrolled and more than 1.5 million cases are being recorded each year. Nationwide data collection and analysis leads to high‐quality health care for patients and the general public.^3^ Since 2014, the NCD has provided a preoperative risk calculator tool that allows clinicians to predict an individual patient's mortality risk for eight surgical procedures.^4^, ^5^, ^6^ Analysis of 19 070 right hemicolectomy cases registered in 2011 indicated that 30‐day mortality (death within 30 days after surgery in or out of hospital) was 1.1% and operative mortality (death within 30 days of surgery or within 90 days of surgery during the same hospitalization) was 2.3%. We previously used the NCD dataset to examine risk models for 30‐day mortalities and operative mortalities after right hemicolectomies;^7^ however, risk models for postoperative morbidity except for operative mortality have not been evaluated until now.

Herein, we report the development and performances of risk models for eight clinical outcomes associated with high mortality or having high incidence after right hemicolectomy, predicting morbidity rate based on preoperative risk factors, using the NCD data from 2011 and 2012.

## 2 MATERIALS AND METHODS

### 2.1 Data source

Japan's National Clinical Database continuously manages registry data by a web‐based data management system, and identifies individuals with annual data approval. We carried out this study using the Japanese Society of Gastroenterological Surgery (JSGS) registry data in the NCD. We enrolled patients who underwent right hemicolectomy between 1 January 2011 and 31 December 2012. A total of 38 924 cases of right hemicolectomy were registered in the NCD during this time. As in our previous study, we excluded 894 cases that had missing values for basic data such as patient gender, age or 30‐day mortality or those that had a simultaneous surgical procedure such as esophagectomy and hepatectomy.^7^ After these exclusions, 38 030 cases that had all the parameters treated in the analyses remained in the study.

The types of data recorded into the JSGS registry in the NCD are almost identical to those used by the ACS NSQIP. Potential independent variables included 15 patient demographic variables, 46 pre‐existing comorbidities, and 19 preoperative laboratory values. Preoperative variables in the NCD are listed in Table S1.

### 2.2 Study outcomes (mortality and postoperative adverse events)

We first assessed the occurrences of the following postoperative adverse event types collected in the NCD database: surgical site infections (SSI) (superficial, deep and organ space), anastomotic leak, pneumonia, urinary tract infection, SIRS (systemic inflammatory response syndrome), sepsis, wound dehiscence, unplanned intubation, pulmonary embolism, renal failure (defined as creatinine elevation over 2 mg/dL of baseline and including acute renal failure treated with filtration or dialysis within 30 days after surgery in a patient without previous dialysis), central nervous system (CNS) occurrences (stroke, coma for more than 24 hours), and cardiac occurrences (cardiac arrest, myocardial infarction, pulmonary embolism) within 30 days after surgery. Among these complications, we selected eight postoperative adverse events for risk modeling analysis based on their high incidence in relation to right hemicolectomy or their strong association with mortality after right hemicolectomy: pneumonia, unplanned intubation, renal failure, CNS occurrences, transfusion >5 units, any systemic sepsis, any SSI, and anastomotic leak. Systemic sepsis includes septic shock, sepsis and SIRS. “Any SSI” was defined to include superficial incisional SSI, deep incisional SSI, organ space SSI, and organ space SSI with leakage. Age (in years), which was one of the predictive factors of postoperative adverse events, was categorized as follows: category 1, <60; 2, 60‐64; 3, 65‐69; 4, 70‐74; 5, 75‐79; 6, 80‐84; 7, 85‐89; 8, 90<. Odd's ratio of age indicates increased risk for one‐category‐up.

### 2.3 Definitions of outcomes

#### 2.3.1 Anastomotic leakage

(ileum‐colon, bile duct‐gastrointestinal trunk, pancreatic duct‐gastrointestinal trunk): When there is drainage from a drain or when drainage is required, or when there is no drainage but it is proven by image.

#### 2.3.2 Superficial incisional SSI

(occurrence within 30 days after surgery): (i) Discharge of pus from the superficial wound part including both confirmation of infection and not); (ii) detection of bacteria by culturing liquids or tissues collected from the superficial wound; (iii) pain, tenderness, swelling, redness and warmth of the wound; (iv) incisional drainage was carried out according to a doctor's diagnosis even if culture negative; (v) diagnosis as SSI by a doctor.

#### 2.3.3 Deep incisional SSI

(occurrence within 30 days after surgery): (i) discharge of pus from deep wound of the muscular fascia or muscle excluding organs or cavity; (ii) spontaneous wound dehiscence occurred, or incisional drainage of muscular layer was carried out according to a doctor's diagnosis as a result of fever over 38°C, pain and tenderness of the wound even if culture negative; (iii) SSI of deep wound infection diagnosed by visible abscess formation, culture inspection, reoperative findings, tissue examinations or imaging study etc.; (iv) diagnosis as deep SSI by a doctor and infection from the superficial to deep wound.

#### 2.3.4 Organ space SSI

(occurrence within 30 days after surgery): (i) drainage from a drain placed in an organ or abdominal cavity or thoracic cavity; (ii) detection of bacteria by culturing liquids or tissues collected from organs or body cavities; (iii) deep wound infection diagnosed by visible abscess formation, culture inspection, reoperative findings, tissue examinations and imaging study etc.; (iv) diagnosis as organ or body cavity SSI by a doctor.

#### 2.3.5 Pneumonia

(occurrence within 30 days after surgery): It is defined as inflammation of the lung caused by bacteria, viruses or chemical irritants (it did not exist before surgery). It develops with cold sweat, fever, chest pain, cough and purulent sputum within 30 days after surgery. Definition of pneumonia requires that one of the following two criteria must be met:

Criterion 1: Chest examination shows pulmonary noise or bluntness of a chest X‐ray and one of the following: (i) newly started purulent sputum or change of sputum aspect; (ii) detection of microorganisms in blood culture; (iii) detection of microorganism from specimen obtained by transbronchial aspiration, bronchial brushing or biopsy.
Criterion 2: Newly progressing infiltration shadow, cavities, pleural effusions in chest X‐ray or any of the following: (i) newly started purulent sputum or change of sputum aspect; (ii) detection of microorganisms in blood culture; (iii) detection of microorganism from specimen obtained by the transbronchial aspiration, bronchial brushing or biopsy; (iv) isolation of virus or detection of viral antigens in respiratory secretions; (v) antibody titer (immunoglobulin [Ig]M) or fourfold increase in paired sera (IgG); (vi) histopathological findings of pneumonia.

#### 2.3.6 Systemic inflammatory response syndrome (SIRS)

Definition of SIRS requires that two of the following criteria must be met: (i) body temperature >38°C or <36°C; (ii) heart rate >90/minute; (iii) respiration rate >20/minute or PaCO_2_ <32 mm Hg; (iv) leukocytes >12 000 cells/mm^3^ or <4000 cells/mm^3^ or bands >10%.

#### 2.3.7 Sepsis

Sepsis shows signs and symptoms of SIRS. Definition of sepsis requires that one of the following criteria must be met: (i) blood culture positive; (ii) proof of maturation or detection of bacterial culture from infectious lesion.

#### 2.3.8 Severe sepsis/septic shock

(i) Organ disorders or circulatory insufficiency; (ii) symptoms of organ disorder include hypouresis, acute changes of mental condition or acute respiratory distress; (iii) symptoms of circulatory insufficiency include hypotension and necessity for cardiotonic agents or vasopressors.

#### 2.3.9 CNS occurrence

(occurrence within 30 days after surgery): Motor and/or sensory disorder as a result of cerebral infarction, thrombosis or hemorrhage, or continuous cognitive disability after surgery.

#### 2.3.10 Renal failure

(occurrence within 30 days after surgery): (i) 2 mg/dL or more than 2 mg/dL increase compared to preoperative value of creatinine without dialysis; (ii) implementation of hemofiltration, hemodialysis or peritoneal dialysis for patients who had no treatment for dialysis (acute renal failure).

### 2.4 Statistical analysis

Pearson's correlation coefficients between each of the eight complications and operative mortality were estimated. Following the definition used in previous studies from NCD, we defined 30‐day mortality as death within 30 days regardless of hospitalization status, and operative mortality as death within 30 days or at discharge.

For risk modeling, we first divided the cohort randomly into an 80% development cohort and a 20% testing cohort, because building a model in a dataset with a large number of outcomes was more important than testing the accuracy (i.e. estimating c‐statistics or depicting receiver operating characteristic [ROC] curves) of the model with greater precision. Within the development cohort, we constructed logistic regression models for the outcomes of interest from the preoperative risk factors listed above, using a forward stepwise selection method with an inclusion criteria of *P* < .05 and an exclusion criteria of *P* ≥ .10. We then applied the models to the patients in the testing cohort, predicting their baseline risk of the outcome using the following equation: Predicted morbidity was calculated as $e^{\left(\beta 0+\sum \beta \mathrm{ixi}\right)}/1+e^{\left(\beta \sum \beta \mathrm{ixi}\right)}$ where βi is the coefficient of variable Xi from the logistic regression model in the development cohort.^4^ Using the predicted risk, we assessed the discrimination of the models using ROC curves and their c‐indices. We used IBM SPSS Statistics for Windows (Version 20; IBM, Armonk, NY, USA) for data analysis.

## 3 RESULTS

The complication of “any SSI” was identified most frequently and its incidence rate was 7.5%. Morbidity rates of other clinically important postoperative outcomes were as follows: anastomotic leakage (1.8%), pneumonia (1.8%), systemic sepsis including septic shock, sepsis and systemic inflammatory response syndrome (2.3%), unplanned intubation (0.8%), renal failure (1.3%), CNS occurrences (0.8%), and transfusion >5 units (1.0%) in the development cohort. Additionally, all of the database's clinical outcomes were listed separately according to operative mortality rates (total number of deaths/total number of cases) for each clinical outcome in the total 38 030 cases. We tabulated the number of patients among all patients in our study cohort having each of the outcomes, and also estimated the proportion of those who died among those with the complications (Table 1). Cardiac occurrences (80.7%), septic shock (60.2%), acute renal failure (57.5%), unplanned intubation (52.2%), CNS occurrences (51.7%), renal failure (46.1%), prolonged ventilation over 48 hours (45.2%), transfusion >5 units (42.1%), and any systemic sepsis (36.1%) showed high operative mortality rates.

**Table 1:** Outcomes of 38 030 right hemicolectomies from 2011 and 2012 NCD data

Variables	No. deaths	Total no. events	Total no. deaths/events (%)	Total no. events/cases (%)
Mortality				
Thirty‐day mortality	438	438	100.0	1.2
Operative mortality	802	802	100.0	2.1
General				
Any complication	584	6384	9.1	16.8
Any transfusion	271	1075	25.2	2.8
Wound‐related				
Superficial incisional SSI	146	2252	6.5	5.9
Deep incisional SSI	108	702	15.4	1.8
Organ space SSI	138	898	15.4	2.4
Wound dehiscence	79	475	16.6	1.2
Anastomotic‐related				
Anastomotic leak	107	696	15.4	1.8
Systemic sepsis				
Septic shock	203	337	60.2	0.9
Sepsis	68	265	25.7	0.7
SIRS	47	278	16.9	0.7
Respiratory events				
Pneumonia	196	690	28.4	1.8
Unplanned intubation	165	316	52.2	0.8
Prolonged ventilation over 48 h	256	566	45.2	1.5
Renal and urinary tract events				
Renal failure	220	477	46.1	1.3
Acute renal failure	130	226	57.5	0.6
Cerebrovascular events				
CNS occurrences	152	294	51.7	0.8
Cardiac events				
Cardiac occurrence	138	171	80.7	0.4
Pulmonary embolism	9	54	16.7	0.1
Reoperation and readmission				
Reoperation and readmission	145	1259	11.5	3.3
Reoperation within 30 d	122	1205	10.1	3.2
Readmission within 30 d	10	1027	1.0	2.7

CNS, central nervous system; SIRS, systemic inflammatory response syndrome; SSI, surgical site infection.

### 3.1 Correlation between mortality and the eight selected postoperative clinical outcomes

Correlation between 30‐day mortality rates and operative mortality rates and the eight selected postoperative clinical outcomes after right hemicolectomy were analyzed using Pearson's correlation coefficient (*r*). Unplanned intubation (*r* = .316), renal failure (*r* = .341), CNS occurrence (*r* = .301) and any systemic sepsis (*r* = .360) were well correlated with operative mortality (Table 2). Additionally, septic shock (*r* = .378), which was included in systemic sepsis, prolonged ventilation over 48 hours (*r* = .365) and cardiac occurrence (*r* = .364) were identified as postoperative clinical outcomes well correlated with operative mortality.

**Table 2:** Correlation between mortality and the 8 selected postoperative morbidities

	Thirty‐day mortality	Operative mortality	Pneumonia	Unplanned intubation	Renal failure	CNS occurr. any	Transfusion >5 units	Systemic sepsis any	SSI any	Anastomotic leak
Thirty‐day mortality	–	0.728	0.201	0.297	0.313	0.286	0.221	0.310	0.050	0.057
Operative mortality		–	0.246	0.316	0.341	0.301	0.281	0.360	0.099	0.124
Pneumonia			–	0.328	0.279	0.202	0.242	0.356	0.148	0.102
Unplanned intubation				–	0.258	0.253	0.295	0.321	0.095	0.145
Renal failure					–	0.314	0.344	0.418	0.146	0.147
CNS occurrences any						–	0.259	0.284	0.076	0.075
Transfusion >5 units							–	0.351	0.125	0.155
Systemic sepsis any								–	0.253	0.299
SSI any									–	0.331
Anastomotic leak										–

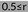
; 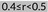; 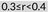.

CNS, central nervous system; SSI, surgical site infection.

### 3.2 Preoperative variables selected for the eight postoperative adverse events

Predictors just before surgery and their estimated odds ratios (OR) (95% CI) from the risk models of the eight selected postoperative clinical outcomes are tabulated in Table 3.

**Table 3:** Logistic regression analysis for the development data set predicting the 8 selected postoperative adverse events

Variables	SSI (n=21)	
	Odd's ratio (95% CI)	*P*‐values
Demographics		
Sex male	1.19 (1.07‐1.31)	.001
Emergent surgery	1.58 (1.16‐1.59)	<.001
ASA grade 3 and over	1.26 (1.12‐1.41)	<.001
BMI over 25	1.44 (1.30‐1.60)	<.001
Comorbidities		
Alcohol	1.10 (1.00‐1.22)	.049
Brinkman index over 400	1.18 (1.06‐1.33)	.004
ADL preope any assistance	1.32 (1.15‐1.50)	<.001
Weight loss over 10 percent	1.25 (1.06‐1.48)	.008
Open wound	2.54 (1.47‐4.38)	.001
COPD	1.47 (1.19‐1.82)	<.001
Ascites	1.35 (1.12‐1.62)	.001
Systemic sepsis	1.41 (1.11‐1.79)	.005
No tumor	1.30 (1.10‐1.53)	.002
Cancer metastasis relapse	1.50 (1.23‐1.83)	<.001
Preoperative laboratory data		
ALB under 2	1.41 (1.07‐1.86)	.015
ALB under 4	1.18 (1.07‐1.30)	.001
ALP over 340	1.26 (1.11‐1.43)	<.001
Na under 138	1.12 (1.01‐1.25)	.039
CRP over 1	1.28 (1.15‐1.43)	<.001
CRP over 10	1.24 (1.03‐1.49)	.021
PT INR over 1.1	1.12 (1.01‐1.25)	.040

Variables	Anastomotic leakage (n=15)	
	Odd's ratio (95% CI)	*P*‐values
Demographics		
Sex male	1.64 (1.38‐1.96)	<.001
ASA grade 3 and over	1.41 (1.15‐1.74)	.001
BMI over 30	1.82 (1.16‐2.87)	.010
BMI over 26	1.36 (1.05‐1.77)	.022
Comorbidities		
Weight loss over 10 percent	1.85 (1.41‐2.44)	<.001
Open wound	2.71 (1.28‐5.76)	.009
COPD	1.48 (1.01‐2.15)	.043
Ascites without control	1.71 (1.25‐2.34)	.001
Peripheral vein disease	2.17 (1.06‐4.47)	.035
No tumor	2.21 (1.74‐2.81)	<.001
Cancer metastasis relapse	1.58 (1.09‐2.28)	.016
Preoperative laboratory data		
ALB under 2	2.11 (1.40‐3.18)	<.001
ALB under 3	1.53 (1.21‐1.93)	<.001
Na under 130	1.61 (1.01‐2.57)	.047
CRP over 1	1.27 (1.04‐1.55)	.017

Variables	Systemic sepsis (n=20)	
	Odd's ratio (95% CI)	*P*‐values
Demographics		
Sex male	1.69 (1.42‐2.01)	<.001
Ambulance transport	1.45 (1.15‐1.84)	.002
Emergent surgery	1.86 (1.46‐2.38)	<.001
ASA grade 5	2.58 (1.58‐4.21)	<.001
ASA grade 3 and over	1.61 (1.32‐1.96)	<.001
Comorbidities		
ADL preope any assistance	1.65 (1.35‐2.02)	<.001
Weight loss over 10 percent	1.50 (1.15‐1.95)	.003
COPD	1.86 (1.35‐2.58)	<.001
Ascites	1.35 (1.04‐1.75)	.025
Systemic sepsis	4.29 (3.30‐5.58)	<.001
No tumor	1.31 (1.03‐1.68)	.031
Preoperative laboratory data		
PLT under 5	2.18 (1.05‐4.51)	.036
ALB under 2	1.61 (1.13‐2.30)	.009
ALB under 3.5	1.59 (1.25‐2.01)	<.001
ALB under 4	1.31 (1.01‐1.70)	.043
ALP over 340	1.28 (1.02‐1.60)	.030
CRP over 1	1.36 (1.12‐1.65)	.002
Tatal bilirubin over 1.2	1.47 (1.13‐1.90)	.004
AST GOT over 35	1.34 (1.07‐1.67)	.010
Urea nitrogen over 20	1.58 (1.31‐1.91)	<.001

Variables	Pneumonia (n=19)	
	Odd's ratio (95% CI)	*P*‐values
Demographics		
Age category 2	1.22 (1.19‐1.32)	<.001
Sex male	2.69 (2.20‐3.28)	<.001
Emergent surgery	1.51 (1.17‐1.94)	.002
ASA grade 4 and 5	1.61 (1.11‐2.34)	.011
ASA grade 3 and over	1.37 (1.10‐1.69)	.004
Comorbidities		
ADL preope any assistance	2.28 (1.84‐2.82)	<.001
Respiratory distress	1.56 (1.15‐2.12)	.004
Preope pneumonia	3.04 (2.05‐4.51)	<.001
COPD	2.01 (1.47‐2.74)	<.001
Ascites	1.45 (1.09‐1.92)	.011
Previous PVD suegery	2.07 (1.10‐3.92)	.024
Systemic sepsis	1.86 (1.32‐2.62)	<.001
Disseminated cancar	1.40 (1.02‐1.90)	.035
Preoperative laboratory data		
PLT under 8	2.15 (1.32‐3.51)	.002
ALB under 3.5	1.59 (1.30‐1.96)	<.001
ALP over 600	1.74 (1.14‐2.64)	.010
CRP over 1	1.34 (1.08‐1.65)	.007
PT INR over 1.1	1.24 (1.02‐1.52)	.033
Urea nitrogen over 20	1.30 (1.06‐1.60)	.013

Variables	Unplanned intubation (n=16)	
	Odd's ratio (95% CI)	*P*‐values
Demographics		
Age category	1.19 (1.09‐1.30)	<.001
Sex male	2.35 (1.77‐3.13)	<.001
Emergent surgery	1.46 (1.02‐2.07)	.037
ASA grade 3 and over	1.78 (1.33‐2.38)	<.001
Comorbidities		
ADL preope any assistance	1.57 (1.16‐2.14)	.004
Respiratory distress	1.68 (1.12‐2.53)	.012
COPD	2.11 (1.37‐3.25)	.001
Previous PVD suegery	2.66 (1.26‐5.62)	.011
Systemic sepsis	2.16 (1.39‐3.37)	.001
Disseminated cancar	1.67 (1.12‐2.50)	.012
Preoperative laboratory data		
PLT under 8	2.29 (1.25‐4.18)	.007
ALB under 3.5	2.40 (1.79‐3.21)	<.001
Na under 130	2.63 (1.60‐4.32)	<.001
PT INR over 1.1	1.42 (1.08‐1.88)	.013
AST GOT over 40	1.45 (1.03‐2.03)	.032
Urea nitrogen over 20	1.48 (1.11‐1.99)	.009

Variables	Renal failure (n=19)	
	Odd's ratio (95% CI)	*P*‐values
Demographics		
Ambulance transport	1.76 (1.31‐2.37)	<.001
Emergent surgery	2.00 (1.44‐2.79)	<.001
ASA grade 4 and 5	1.45 (0.99‐2.10)	.054
ASA grade 3 and over	1.94 (1.48‐2.54)	<.001
BMI over 30	3.14 (1.15‐8.58)	.025
Comorbidities		
ADL preope any assistance	1.55 (1.20‐2.01)	.001
Preoperative ventilation	1.85 (1.03‐3.33)	.040
Hypertention	1.34 (1.07‐1.69)	.012
Ascites	1.52 (1.10‐2.11)	.012
Systemic sepsis	1.51 (1.06‐2.15)	.023
No tumor	1.42 (1.02‐1.97)	.036
Disseminated cancar	2.29 (1.60‐3.28)	<.001
Preoperative laboratory data		
HCT under 30	1.36 (1.06‐1.73)	.015
PLT under 8	1.84 (1.11‐3.07)	.019
ALB under 3.5	1.40 (1.08‐1.80)	.010
ALP over 600	2.53 (1.61‐3.99)	<.001
Tatal bilirubin over 1.2	1.75 (1.26‐2.43)	.001
Urea nitrogen over 20	2.09 (1.56‐2.79)	<.001
Creatinine over 1.2	3.41 (2.57‐4.52)	<.001

Variables	CNS occurrence (n=18)	
	Odd's ratio (95% CI)	*P*‐values
Demographics		
Age	1.01 (1.00‐1.03)	.035
Emergent surgery	1.64 (1.08‐2.48)	.020
ASA grade 4 and 5	1.82 (1.14‐2.88)	.012
ASA grade 3 and over	1.74 (1.24‐2.44)	.001
BMI over 30	2.51 (1.26‐5.01)	.009
Comorbidities		
ADL preope any assistance	2.39 (1.73‐3.29)	<.001
Preoperative ventilation	3.14 (1.67‐5.92)	<.001
Hypertention	1.51 (1.13‐2.01)	.006
Previous PVD suegery	3.94 (1.92‐8.08)	<.001
Cerebrovascular disease	2.47 (1.72‐3.55)	<.001
Systemic sepsis	2.01 (1.30‐3.10)	.002
No tumor	1.81 (1.18‐2.78)	.007
Disseminated cancar	1.74 (1.10‐2.75)	.018
Preoperative laboratory data		
ALB under 3.5	1.79 (1.30‐2.47)	<.001
ALP over 600	3.09 (1.85‐5.18)	<.001
Na under 138	1.39 (1.03‐1.88)	.032
Tatal bilirubin over 2	2.44 (1.41‐4.23)	.001
Creatinine over 1.2	1.86 (1.34‐2.59)	<.001

Variables	Transfusion > 5U (n=20)	
	Odd's ratio (95% CI)	*P*‐values
Demographics		
Sex male	1.84 (1.41‐2.40)	<.001
Ambulance transport	1.42 (1.02‐1.97)	.036
Emergent surgery	2.06 (1.48‐2.86)	<.001
ASA grade 5	2.88 (1.65‐5.01)	<.001
ASA grade 3 and over	2.73 (2.04‐3.65)	<.001
BMI over 30	2.28 (1.19‐4.37)	.013
Comorbidities		
ADL preope any assistance	1.61 (1.21‐2.15)	.001
Weight loss over 10 percent	1.62 (1.11‐2.37)	.013
Preoperative ventilation	3.06 (1.72‐5.44)	<.001
Ascites	1.95 (1.30‐2.71)	<.001
Systemic sepsis	1.53 (1.05‐2.24)	.026
Preoperative laboratory data		
HCT under 30	1.44 (1.10‐1.90)	.008
PLT under 5	4.47 (1.12‐5.46)	.026
PLT under 15	2.16 (1.57‐2.97)	<.001
ALB under 3	1.76 (1.32‐2.35)	<.001
ALP over 600	2.19 (1.35‐3.54)	.001
Na under 130	1.78 (1.07‐2.96)	.008
PT INR over 1.1	1.61 (1.24‐2.11)	<.001
Tatal bilirubin over 1.2	1.80 (1.28‐2.53)	.001
Urea nitrogen over 25	1.83 (1.36‐2.54)	<.001

Twenty‐one factors were selected as risk factors in the model for any SSI. Among the independent risk factors for SSI, open wound alone was the variable with OR >2 (OR, 2.54), and this risk model of SSI consisting of preoperative factors did not predict well.

Fifteen factors were selected as risk factors in the model for anastomotic leakage. Those with OR >2 were peripheral vascular disease (PVD; OR, 2.17), open wound (OR, 2.71), no tumor (OR, 2.21) and albumin <2 mg/dL (OR, 2.11) as shown in Table 3. Although open wound was one of the risk factors for SSI and anastomotic leakage, the number of patients with an open wound was limited to 0.2% (90 out of 38 030 cases). This risk model of anastomotic leakage also could not predict well.

Risk models of the six life‐threatening postoperative adverse events tended to consist of risk factors related to demographics and comorbidities with higher OR than preoperative laboratory data.

Twenty factors were selected as risk factors for postoperative systemic sepsis. Those with OR >2 were systemic sepsis just before surgery (OR, 4.29), American Society of Anesthesiologists (ASA) grade 5 (OR, 2.58) and platelet level <50 000/μL.

Nineteen factors were selected as risk factors for pneumonia. Those with odds ratios >2 were male gender (OR, 2.69), needing any assistance with activities of daily living (ADL) before surgery (OR, 2.28), chronic obstructive pulmonary disease (COPD) (OR, 2.01), history of pneumonia before surgery (OR, 3.04), previous PVD surgery (OR, 2.08) and platelet levels <80 000/μL (OR, 2.15).

Sixteen factors were selected as risk models for unplanned intubation. Those with OR >2 were patients’ gender being male (OR, 2.35), COPD (OR, 2.11), previous PVD surgery (OR, 2.66), systemic sepsis just before surgery (OR, 2.16), platelet levels <80 000/μL (OR, 2.29), albumin <3.5 (OR, 2.40), and Na <130 mEq/L (OR, 2.63).

Nineteen factors were selected as risk factors for renal failure. Those with OR >2 were disseminated cancer (OR, 2.29), body mass index (BMI) >35 (OR, 3.14), creatinine >1.2 mg/dL (OR, 3.41), alkaline phosphatase (ALP) >600 U/L (OR, 2.53) and urea nitrogen >20 mg/dL (OR, 2.09).

Eighteen factors were selected as risk factors for CNS occurrences. Those with OR >2 were any assistance with ADL before surgery (OR, 2.39), preoperative ventilation (OR, 3.14), previous PVD surgery (OR, 3.94), cerebrovascular disease (OR, 2.47), preoperative systemic sepsis (OR, 2.01), BMI >30 (OR, 2.51), total bilirubin >2 mg/dL (OR, 2.44) and ALP >600 U/L (OR, 3.09).

Twenty factors were selected as risk factors for transfusion >5 units. Those with OR >2 were emergency surgery (OR, 2.06), preoperative ventilation (OR, 3.06), ASA grade 5 (OR, 2.88), ASA ≥grade 3 (OR, 2.73), BMI >30 (OR, 2.28), platelets <150 000/μL (OR, 2.16), platelets <50 000/μL (OR, 2.47) and ALP >600 U/L (OR, 2.19).

### 3.3 Model performance

**Figure 1:**
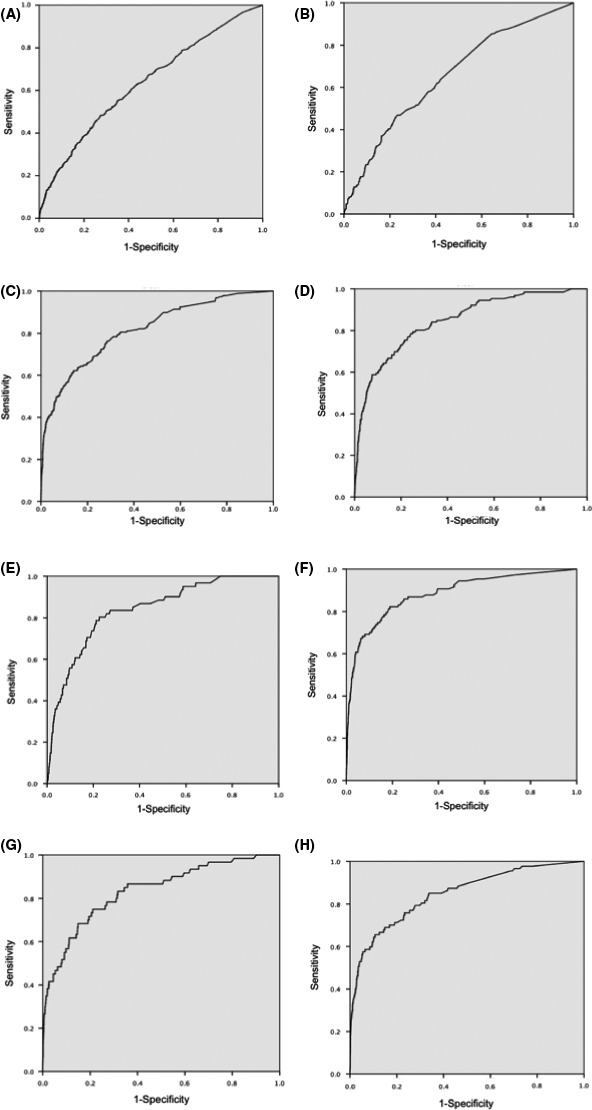
Receiver operating characteristics curve for each clinical outcome in this risk model. A, Surgical site infection; B, anastomotic leak; C, systemic sepsis; D, pneumonia; E, unplanned intubation; F, renal failure; G, central nervous system occurrence; H, transfusion >5 units

Concordance indices (C‐indices) of the risk models for these major postoperative morbidities estimated in the testing samples (n = 7423) are summarized in Table 4, and the resultant eight ROC curves are shown in Figure 1. The models for sepsis, pneumonia, unplanned intubation, renal failure, CNS occurrence, and transfusion >5 units had good discriminatory powers, whereas those for SSI and anastomotic leak were less satisfactory.

**Table 4:** Evaluation of risk models of the eight selected clinical outcomes after right hemicolectomy in the testing sample (n = 7423)

	C‐index	95% CI	*P*‐value
SSI	0.634	0.610‐0.659	<.0001
Anastomotic leakage	0.656	0.608‐0.704	<.0001
Systemic sepsis	0.816	0.782‐0.850	<.0001
Pneumonia	0.846	0.810‐0.882	<.0001
Unplanned intubation	0.838	0.789‐0.887	<.0001
Renal failure	0.883	0.846‐0.921	<.0001
CNS occurrence	0.833	0.776‐0.890	<.0001
Transfusion >5 units	0.846	0.799‐0.892	<.0001

Systemic sepsis, septic shock; sepsis; SIRS.

C‐index, concordance index; CNS, central nervous system; SSI, surgical site infection.

## 4 DISCUSSION

The NCD of Japan was established in April 2010; since then, more than 4600 facilities have enrolled and more than 1.5 million cases covering approximately >95% of all general surgical procedures are being enrolled each year. Typically, such nationwide databases include data on 30‐day mortality and operative mortality,^4^, ^7^ and this Japanese database provided data showing that the overall 30‐day mortality and operative mortality rates after right hemicolectomy were 1.1% and 2.3%, respectively,^7^ and that the incident rates were 1.2% and 2.2%, respectively, confirming previous reports. The present study developed and assessed risk models for eight postoperative clinical outcomes selected for their high frequency or high lethality in relation to right hemicolectomy. The preoperative variables were selected from all preoperative variables including patient demographics, pre‐existing comorbidities/disease histories and preoperative laboratory values using a forward stepwise selection corresponding to each clinical outcome after right hemicolectomy. The result is a risk calculator to predict clinical postoperative complications based on a patient's preoperative factors.

The incident rates of the eight selected clinical outcomes (incident rates) were SSI (7.5%), anastomotic leakage (1.9%), pneumonia (1.8%), systemic sepsis (2.5%), unplanned intubation (0.8%), renal failure (1.2%), CNS occurrence (0.8%) and transfusion >5 units (1.0%) based on the development sample of patients (n = 30 607) randomly selected from this study. ROC curves and concordance indices for the remaining sample of patients (n = 7423) showed that the developed risk models for six of the eight examined clinical outcomes—namely, the six mortality‐associated outcomes—were accurate predictive tools. Although the accuracy of prediction for SSI or anastomotic leakage did not reach the level of those for the morbidities associated with mortality, the models suggested the relation of certain preoperative comorbidities and laboratory data to the occurrences of SSI and anastomotic leakage, which hamper early discharge of patients and are a significant source of aggravation to surgeons and patients alike. We suspected that these results indicated that not only preoperative factors but also intraoperative factors influenced this risk model of SSI or anastomotic leakage such as non‐life‐threatening postoperative complications.

There have been various studies of risk stratification of mortality and/or morbidities using a nationwide database after surgery.^8^, ^9^ Recently, the Surgical Risk Preoperative Assessment System (SURPAS) showed that accurate preoperative risk assessment of postoperative mortality, overall morbidity, and six complication clusters in a broad surgical population could be achieved with as few as eight preoperative predictor variables.^9^ However, the authors pointed out some limitations because as they cover a broad spectrum of operations carried out by nine surgical specialties (general, vascular, orthopedic, otorhinolaryngological, urological, thoracic, plastic, gynecological, and neurosurgery), generic predictors and outcome variables were necessarily chosen rather than more operation‐ or disease‐specific ones. They also mentioned that these specific complications might be more closely correlated with the occurrence of one of the 18 NSQIP complications. A more specific examination for colectomy is exactly what we have done in the present study. We could show preoperative comorbidities and laboratory data for lethal complications and surgically related complications (SSI and anastomotic leakage) of this procedure.

We found that the following variables are similarly highly predictive across models for different lethal morbidities: emergent surgery, ASA grade ≥3, poor ADL and preoperative systemic sepsis. These observations were compatible with previous findings by Meguid et al^9^ or Cohen et al,^10^ both of whom used NSQIP data for analysis. We were able to analyze laboratory data (low serum albumin and low renal function parameters) as potential risk variables for each morbidity in this study. Zielsdorf et al reported that model for end‐stage liver disease (MELD) was important for perioperative outcomes in general surgery,^11^ and Causey et al^12^ reported that MELD‐Na was important for morbidity and mortality following elective colon cancer surgery. Other relevant variables (T‐bilirubin, creatinine and prothrombin time‐international normalized ratio [PT‐INR]) were chosen as predictors for certain morbidities in this study.

When we discuss SSI, we need to pay attention to differences in risk factors for individual types of SSI as mentioned by Segal et al.^13^ They reported that organ‐space SSI were often caused by anastomotic leaks and were uniquely associated with disseminated cancer, preoperative dialysis, preoperative radiation treatment, and bleeding disorders, suggesting that a physically frail or compromised patient may encounter greater risk to an anastomotic leakage. In our study, male gender, ASA ≥3, weight loss, open wound, relapse of cancer metastasis and laboratory data including low serum albumin, low Na level and elevated C‐reactive protein (CRP) levels were identified as predictors. These parameters were quite consistent with the physically frail health of patients developing anastomotic leakage. There have been many trials to reduce SSI or anastomotic leakage based on the concept of giving antibiotics and surgical approach (open vs laparoscopic).^14^, ^15^, ^16^, ^17^ These effects could also be evaluated in Japanese patients by risk‐adjusted analysis using the risk models created in this study. Based on the concept of the risk model, we can share the incident rates about postoperative complications with medical staff under intensive perioperative management, and we can also consider surgical procedure instead of right hemicolectomy in order to reduce the risks of mortality and morbidity. Furthermore, it is possible (but not for emergency patients) to prepare preoperative conditions on preoperative risk factors shown in Table 3.

Our study has some important limitations to consider. First, emergency surgery, especially for those in the septic condition, has great impact on the mortality and morbidity of patients. Mortality and morbidity are typically high in such patients compared to those undergoing elective surgery. In this analysis, patients who were diagnosed with acute diffuse peritonitis were categorized as critically ill patients undergoing surgery, and risk models for these patients were created separately as reported previously.^18^ Second, the risk models created in the present study were based strictly on Japanese patients. In the previous collaborative study between NSQIP and NCD, we found that patient background, comorbidities, and practice style in Japan and the USA had key differences.^8^ In the models, the OR for each variable was similar between NCD and ACS‐NSQIP, but some risk predictors were population specific. The generalizability of these models to patients in other countries needs to be further investigated in future studies. Future cross‐population evaluations are expected to help prevent complications and reduce health‐care spending.^19^

Risk models of postoperative outcomes in the present study were analyzed using only preoperative variables. In contrast, intraoperative factors influence postoperative outcomes. Risk assessment including intraoperative variables will be evaluated in a future study from the NCD.

In conclusion, we analyzed the performance of risk models for the eight most serious and/or common postoperative adverse events after right hemicolectomy. This study showed that the NCD‐generated risk models, especially for the six lethal postoperative outcomes examined, worked well in discriminating patients who develop these events after right hemicolectomy surgeries. These risk models will greatly enhance the evaluation of individual patients prior to surgery.

## DISCLOSURE

Funding: This study was supported by a research grant from the Ministry of Health, Labour and Welfare in Japan.

Conflicts of Interest: Author H.K. is affiliated with the Department of Healthcare Quality Assessment at the University of Tokyo which is a social collaboration department supported by National Clinical Database. The department was formerly supported by endowments from Johnson & Johnson K.K., Nipro Co., Teijin Pharmaceutical Co., Ltd, W.L. Gore & Associates, Co., Ltd, Olympus Corporation, and Chugai Pharmaceutical Co., Ltd.
