# Supplementary material for: Risk assessment of morbidities after right hemicolectomy based on the National Clinical Database in Japan
Source: Ann Gastroenterol Surg. 2018 Apr 16;2(3):220–30. doi: 10.1002/ags3.12067 (PMC5980426; doi:10.1002/ags3.12067)
Supplement: Supplementary file 1 [file AGS3-2-220-s001.docx]

| Supplemental Table 1 The list of preoperative variables |
| --- |
| Patient demographics (15 variables) |
| Identification code |
| Initials of patient's name |
| Date of birth (Age)  Category: 1, <60; 2, 60-64; 3, 65‐69; 4, 70‐74; 5, 75‐79; 6, 80‐84; 7, 85‐89; 8, 90< |
| Sex male |
| Nationality |
| Refusal of registration |
| Date of admission |
| Ambulance transport |
| Postal code of home address |
| Postal code of address on emergency |
| Diagnosis on admission |
| Height (cm) |
| Weight (kg) |
| BMI >25, >30 |
| BSA (m2) |
| Pre-existing comorbidities (46 variables) |
| Emergency surgery |
| Date of operation |
| Preoperative chemotherapy |
| Preoperative radiotherapy |
| Preoperative immunotherapy |
| Other medication prior to operation within 90 days |
| Diabetes mellitus |
| Smoking (within a year) |
| Smoking history (Brinkman index > 400) |
| Alcoholism |
| Respiratory distress within 30 days |
| ADL preoperative any assistance |
| COPD |
| Pneumonia on admission |
| Encephalopathy within 30 days |
| Esophageal varices any |
| Esophageal varices without control |
| Ascites within 30 days |
| Ascites without control |
| Hypertension within 30 days |
| Hypertension without treatment |
| Congestive heart failure within 30 days |
| Myocardial infarction within 6 months |
| Angina within 30 days |
| Previous PCI |
| Previous cardiac surgery |
| Peripheral vein disease surgery |
| Symptom of peripheral vein disease |
| Acute renal failure within 24 hours |
| Preoperative dialysis within 14 days |
| Cerebrovascular disease |
| Cerebrovascular disease within 14 days |
| Cancer with multiple metastasis |
| Open wound |
| Chronic use of steroid |
| Weight loss over 10 percent |
| Bleeding disorder just before surgery |
| Preoperative blood transfusion within 72 hours |
| Chemotherapy within 30 days |
| Sepsis just before surgery |
| Systemic Inflammatory Response Syndrome |
| Sepsis |
| Septic shock |
| Any surgery except for local anesthesia |
| ASA grade3 and over, 3 and 4, >5 |
| No tumor (No malignancy) |
| Preoperative laboratory values (19 variables) |
| White blood cell <3500, >9000, >11000, >12000 (/μl) |
| Hemoglobin <13.5 in male and <12.5, <10, <7 (g/dl) |
| Hemoglobin >17 in male and >15 in female (g/dl) |
| Hematocrit <37% in male and <32% in female |
| Hematocrit <30, <21 |
| Platelet <5, 8, 12, 15 (X10, 000/μl) |
| Platelet >35, >40 (X10, 000/μl) |
| Total bilirubin <0.2 (mg/dl) |
| Total bilirubin >1.2, >2.0, >3.0 (mg/dl) |
| AST <10 (U/l) |
| AST >35, >40, >100 (U/l) |
| ALT <0 (U/l) |
| ALT >35, 100 (U/l) |
| ALP <110 (IU/l) |
| ALP >340, >600 (IU/l) |
| Blood urea nitrogen <8 (mg/dl) |
| Blood urea nitrogen >20, >25, 40, 60 (mg/dl) |
| Creatinine >1.2, >2.0, >3.0 (mg/dl) |
| ALB <2.0, <2.5, <3.5, <3.8, <4.0 (g/dl) |
| ALB >5.0 (g/dl) |
| eGFR<30 (ml/min) |
| Na <120, <130, <135, <138 (mEq/l) |
| Na >146 (mEq/l) |
| HbA1c >6.5, >7.0, >7.5, >8.0 (%) |
| CRP >1, >10 (mg/dl) |
| PT <10 (sec) |
| PT >50 (sec) |
| PT INR <0.9 |
| PT INR >1.1, >1.25, 1.67 |
| APTT <30 (sec) |
| APTT >40 (sec) |
| ICG15R (%) |
